# Retraction Note: REST regulates the cell cycle for cardiac development and regeneration

**DOI:** 10.1038/s41467-024-46055-8

**Published:** 2024-02-22

**Authors:** Donghong Zhang, Yidong Wang, Pengfei Lu, Ping Wang, Xinchun Yuan, Jianyun Yan, Chenleng Cai, Ching-Pin Chang, Deyou Zheng, Bingruo Wu, Bin Zhou

**Affiliations:** 1 Department of Genetics, Albert Einstein College of Medicine, Bronx, NY 10461 USA; 2 Department of Medical Ultrasound, The First Affiliated Hospital of Nanchang University, Nanchang, 330006 China; 3 Department of Developmental and Regenerative Biology, The Black Family Stem Cell Institute, and The Mindich Child Health and Development Institute, Icahn School of Medicine at Mount Sinai, New York, NY 10029 USA; 4 Department of Medicine, Indian University School of Medicine, Indianapolis, IN 46202 USA; 5 Departments of Genetics, Neurology and Neuroscience, Albert Einstein College of Medicine, Bronx, NY 10461 USA; 6 Departments of Genetics, Pediatrics, and Medicine (Cardiology), The Wilf Cardiovascular Research Institute, The Institute for Aging Research, Albert Einstein College of Medicine, Bronx, NY 10461 USA; 7 Department of Cardiology of First Affiliated Hospital and State Key Laboratory of Reproductive Medicine, Nanjing Medical University, Nanjing, Jiangsu 210029 China

Retraction to: *Nature Communications* 10.1038/s41467-017-02210-y, published online 07 December 2017

The authors have retracted this article because of significant concerns regarding a number of figures presented in this work that question the integrity of the data. After publication, several concerns were raised about the figures in this article.

Specifically,

- There appears to be a partial overlap between two panels of Figure 4e (bottom left corner for p21KO and top right for DKO).
- There appears to be an overlap between a control panel from figure 2k and Rest imKO in Figure 5g (PH3 staining).
- There appears to be image reuse between two samples in Figure 5g in the Aurora B staining row for Rest imKO and p21KO.
- There appears to be an overlap between Figure 6f Ph3 staining for the Rest cDNA sample and Supplementary Fig. 6e, EdU staining, Rest cDNA, with fewer arrows and less visible DAPI staining.

All authors agree with this retraction.

